# On the reduction of mixed Gaussian and impulsive noise in heavily corrupted color images

**DOI:** 10.1038/s41598-023-48036-1

**Published:** 2023-11-29

**Authors:** Bogdan Smolka, Damian Kusnik, Krystian Radlak

**Affiliations:** 1 Faculty of Automatics, Electronics and Computer Science, Silesian University of Technology, Akademicka 16, 44-100 Gliwice, Poland; 2 Institute of Computer Science, Warsaw University of Technology, Nowowiejska 15/19, 00-665 Warsaw, Poland

**Keywords:** Computer science, Information technology, Software

## Abstract

In this paper, a novel approach to the mixed Gaussian and impulsive noise reduction in color images is proposed. The described denoising framework is based on the Non-Local Means (NLM) technique, which proved to efficiently suppress only the Gaussian noise. To circumvent the incapacity of the NLM filter to cope with impulsive distortions, a robust similarity measure between image patches, which is insensitive to the impact of impulsive corruption, was elaborated. To increase the effectiveness of the proposed approach, the blockwise NLM implementation was applied. However, instead of generating a stack of output images that are finally averaged, an aggregation strategy combining all weights assigned to pixels from the processing block was developed and proved to be more efficient. Based on the results of comparisons with the existing denoising schemes, it can be concluded that the novel filter yields satisfactory results when suppressing high-intensity mixed noise in color images. Using the proposed filter the image edges are well preserved and the details are retained, while impulsive noise is efficiently removed. Additionally, the computational burden is not significantly increased, compared with the classic NLM, which makes the proposed modification applicative for practical image denoising tasks.

## Introduction

Image enhancement is one of the most frequently explored topics in image processing and computer vision. Despite the fact that the improvement of image quality has been the object of intensive research for decades, its importance is not weakened. On the contrary, the widespread availability of inexpensive devices that enable the acquisition of visual information makes that methods of image quality enhancement are still in the sphere of active research interest.

The quality of images can be degraded during the acquisition and transmission process. It might be also affected by poor lighting and unfavorable weather conditions. In particular, noise affecting the image capture, due to small sensor size and short time of exposure, reduces the perceptual quality of visual information and impedes diverse image processing tasks. In the rich body of literature, the researchers generally focus on two types of noise models. The first one is the *impulsive noise*, which is caused by malfunctioning camera sensors, electromagnetic interference, aging of the storage material or transmission errors^1–3^. Basically, two models of impulsive noise are considered^4–7^. The first one is *salt and pepper* noise or *fixed value impulse noise*, in which the channel values of corrupted pixels are set to either the minimum or maximum of the dynamic range. The second, much more challenging, is the Random Valued Impulsive Noise (RVIN) noise model, in which color pixel channels may attain any random value within the available range. Color images can be also affected by impulsive noise, which is introduced deliberately forming the adversarial attacks significantly decreasing the efficiency of neural networks, which are widely used for various computer vision tasks^8–10^. The defense techniques, which include image denoising, have recently attracted much attention from researchers working in the field of machine learning and cybersecurity.

The second type of the most frequently encountered image distortion is due to the Gaussian noise arising during acquisition and caused by the intrinsic thermal and electronic fluctuations in camera sensors. This type of noise is evenly distributed over the image and each pixel is distorted by adding a value drawn from a zero-mean Gaussian distribution denoted as Additive White Gaussian Noise (AWGN). The suppression of impulsive and Gaussian noise is extensively covered in the literature, but the reduction of their mixture is less investigated, despite being a more realistic noise model. Since the majority of the widely used denoising algorithms are constructed to exclusively suppress a single type of image contamination, attenuating mixed noise is a difficult undertaking mainly due to the problems with modeling of its distribution^11^. The techniques that can eliminate impulsive noise are ineffective when applied to distortions with Gaussian distribution, but the contrary is also true.

In the literature two main groups of methods for Mixed Gaussian and Impulsive Noise (MGIN) removal are proposed. The straightforward methods of enhancing the noisy image first remove the impulses and then, in the second step, an appropriate filter, designed to reduce the Gaussian noise, is used. Although frequently successful, this strategy has severe drawbacks. The effectiveness of two-step process depends on selecting the appropriate filters and correctly adjusting their parameters, which are often influenced by the noise mixture composition and its intensity. Due to the masking effect, which affects the detection of impulsive noise, and the introduced pixel alterations which go against the assumption of Gaussian noise distribution, sufficient effectiveness of the combined approaches is not guaranteed, despite the increased computational burden.

Consequently, substantial effort has gone into developing reliable filtering techniques that are designed to complete image denoising in a single step. Most of the elaborated approaches are built on established methods that have been shown effective in reducing Gaussian or impulsive noise. These methods are then extended to make them capable of handling both types of disturbances simultaneously.

In this work, an efficient technique capable of suppressing the MGIN is introduced. The proposed technique examines the distances to a prespecified number of similar pixels in the central patch of the processing block and builds a cumulated measure of similarity of the compared patches. In this way, a set of pixels in a patch, which are most similar to the central one in the block, are chosen, thus limiting the risk that impulsive pixels will be used for determining the patches closeness measure.

Following the NLM blockwise processing scheme, we discard the pixels which are not among the most similar ones, assuming that they are corrupted, consequently diminishing the influence of impulsive noise. As a result, from each patch in the processing block we select a group of pixels which are most similar to the central patch and the corresponding pixels from this patch are assigned weights needed for the averaging process. As the positions of the pixels chosen in each patch of the block are changing, every corresponding pixel in the central patch, including the severely corrupted, obtains large number of weighting coefficients, allowing for its robust estimation. The core of the proposed approach is a novel measure of similarity between pixel patches which yields satisfying denoising results.

In the classic blockwise NLM algorithm, each pixel in a patch obtains its estimates which are then averaged in the final denoising step, so that the final output is the mean of a stack of images whose number is equal to the number of pixels in the image patches. In this work, a different strategy, assigning the pixel weights and aggregating them in the final processing stage, without creating separate denoised images, is adopted. Such an approach, proved more robust as the small weights assigned to dissimilar patches are practically discarded in the averaging step. Additionally, performing single weighted averaging saves memory resources as the intermediate results obtained for each position of the central patch do not need to be stored to obtain the final denoising outcome.

The comparison of the efficiency of the proposed robust approach with state-of-the-art denoising schemes confirmed its satisfying properties both in terms of objective restoration measures and visual quality. The proposed filter, due to the properties of the introduced robust measure of similarity across patches, is especially efficient when restoring heavily distorted images. The performed experiments show that setting the filter’s parameters is quite intuitive and its time efficiency is comparable with the standard NLM and even lower for high noise intensity. Additional computational burden is caused mainly by the need of choosing the pixels from a patch which are most similar to the one in block center. However, in practical applications significantly larger blocks and patch sizes are needed by the NLM, which translates into higher computational efficiency.

The remainder of the paper is structured as follows. The next Section presents a brief overview of the rich body of related literature. Then, in section “Robust method of MGIN reduction” the structure of the classic NLM is described. Afterwards, the robust measure of similarity between patches is introduced and ultimately the proposed MIxed NOise Reduction (MINOR) filter is described. In section “Filtering results” the influence of the filter parameters on its denoising efficiency is analyzed and the recommendations regarding their settings are provided. Eventually, the elaborated method is compared with a set of filters intended for the suppression of MGIN and the results are summarized using the standard objective measures of image quality enhancement. Examples of the restoration results obtained with the proposed and competitive techniques allow to visually asses the merits of the proposed denoising scheme. Finally, in section “Conclusion” conclusions are drawn and prospects of improvements which can increase the effectiveness of the proposed denoising framework are outlined.

## Related work

### Impulsive noise removal

The most popular filtering techniques intended for the suppression of impulsive noise are based on the concept of multivariate order statistics^4,12,13^. The widely used Vector Median Filter (VMF), determines the pixel from a local neighborhood for which the sum of distances to all neighbors is minimized^14^. This filter removes effectively the impulsive pixels, however one of its drawbacks is that its output is always one of the pixels from the filtering window and when the image is distorted by MGIN, the Gaussian component is not suppressed.

To diminish the number of unnecessarily processed pixels, the concept of the weighted median^15,16^ was applied and to increase the capacity of VMF to suppress the Gaussian noise the average of the cluster of most similar samples was used as filter output^17,18^. A generalization of VMF, which calculates the cumulated distances to the closest pixels belonging to the filtering window and sharpens image edges, was described in Lukac et al.^19^. The VMF was also extended using the fuzzy similarity measures for vector ordering^20–23^. A detailed study and experimental comparisons of filters based on order statistics intended for impulsive noise suppression can be found in Celebi et al.^21^.

In order to prevent unnecessary processing of undistorted pixels, various switching strategies are employed. Their goal is to detect the corrupted pixels and replace them with an estimate based on the local neighborhood, while keeping the remaining ones unchanged. The switching methods often rely on the vector ordering concept and determine the corrupted pixels calculating their distance to the output of a VMF based filter^24–26^. To tackle the problem of impulsive noise detection, various approaches based on fuzzy logic were elaborated. In^27^, the outlier detection scheme, which compares the central pixel with its neighbors was designed. Then a weighted averaging scheme incorporating the measure of pixel impulsiveness is used to suppress the noisy pixel. A fuzzy-based switching technique for impulse detection and removal builds membership functions based on local pixel similarity to its neighborhood and the results of median based filtering^28–31^.

One of the most efficient filters used for the removal of impulsive noise is the Peer Group Filter (PGF) proposed in Kenney et al.^32^. This technique calculates the distances between the central pixel of the filtering window and its neighbors, which are later sorted to find the set of the closest pixels called a *peer group*. Another approach is determining the number of pixels in the filtering window whose distance does not exceed a predefined threshold^33^. The peer-group based techniques were later extended, however, likewise other switching filters, they remove only outlying pixels and are not effective when dealing with mixed noise^34–36^. In^37^, a novel variational approach to restore color images affected by impulse noise was introduced. The key innovation lies in the adaptive weighting of a data-fidelity term within the cost function. This term, derived using statistical methods, comprises two weighting functions and statistical control parameters for noise. Then a Bayesian framework is formulated where likelihood functions are defined by a mixture model.

### Gaussian noise reduction

The traditional methods of the restoration of color images contaminated with Gaussian noise are based on local averaging. The important drawback of these filters is their inability to suppress the impulsive distortions, which are treated as image structures and are preserved.

The Bilateral Filter (BF), which is highly efficient in suppressing the Gaussian noise^38^ builds the weighted average of pixels belonging to a processing block, considering their topographic closeness and radiometric similarity. However, it assigns the highest value to the central pixel and when it is corrupted, the filter tends to preserve it. Similar problem affects the Anisotropic Diffusion, which calculates the differences between the central pixel of a window and its members^39^ to perform the diffusion process. When the central pixel is an outlier, then all the gradient magnitudes are high and the anisotropic smoothing is terminated, which again preserves the impulses. Other approaches based on Total Variation regularization^40^, which well preserve image edges, wavelet thresholding performing the shrinkage of the corrupted wavelet coefficients^41–43^ and fuzzy-based filters^44,45^ face the same problem.

The powerful Non-local Means (NLM) technique^46,47^ utilizes the image self-similarity property and the final pixel estimation is the weighted average of all pixels which form similar patterns in the patches of the processing block. As this method yields excellent denoising results, an extension based on Block-Matching and 3D filtering (BM3D) exploiting the image local sparse representation in the transform domain was proposed^7,48–50^. These filters provide very satisfying results when the Gaussian noise is encountered, however, they are very sensitive to impulsive noise as the utilized similarity measures between groups of pixels (patches) are susceptible to the impact of outlying pixels and the filters tend to preserve impulsive distortions.

In^51^, a pixel-level Non-Local Self Similarity (NSS) prior was introduced, wherein similar pixels across a non-local region are sought. This approach is motivated by the feasibility of identifying closely matching pixels in natural images. The introduced pixel-level NSS prior allows for an accurate noise level estimation method and the development of a blind image denoising technique based on the lifting Haar transform and Wiener filtering methods.

### Mixed noise reduction

The most straightforward way of dealing with mixed noise is to classify the image pixels as either corrupted by impulsive or Gaussian noise and to restore these pixels with appropriate filters. In^52^, a Support Vector Machine (SVM) classifier is used to detect the type of noise corrupting each pixel to select the median or bilateral filters which suppress the impulses and the Gaussian noise component. Similar approach based on SVM was used in^53^ to choose between NLM and BM3D when enhancing images with noise simulating the distortions typically encountered in natural images.

In^54^, a switching BF was proposed to reduce the mixed noise. First, the pixels are classified, either as impulses, corrupted by Gaussian noise, or noise-free, using a median based technique and then the corrupted pixels are restored by filters, whose structures are controlled by the result of the classification step. A switching BF with a novel weighting function was also used in^55^ to suppress mixed noise in color images. The reference pixel of the BF is replaced by the VMF output when an impulse is detected, otherwise the standard BF is applied.

Another approach^56^ first detects the impulsive noise and replaces the corrupted pixels with the local median. In the next step all pixels are processed using a fuzzy scheme relying on the values of a modified Rank Ordered Absolute Difference, (ROAD). The ROAD statistic^57^, which determines the sum of distances between a pixel and its most similar neighbors from a filtering window, was combined with the fuzzy peer group concept in Camarena et al.^58^. The technique presented in^59^ first identifies impulses using the Adaptive Median Filter (AMF) and subsequently employs a variational step to eliminate residual noise. Experimental results indicate that integrating this approach with established methods can substantially enhance their denoising effectiveness In^60^, the fuzzy peer groups were used in a two-step filter which cascades the impulse removal with fuzzy averaging, performed on the same cluster of similar pixels, which reduces the computational complexity. A fast parallel implementation of the fuzzy peer group processing was put forward in Arnal et al.^61^.

Many techniques attempt to adopt patch-based methods to deal with mixed noise. However, first the impulsive noise must be removed to enable the reduction of Gaussian noise in the subsequent denoising step. In^62^, the impulses were detected and suppressed using the the median filter and a robust variance measure and afterwards the BM3D technique was applied. Similar approach was presented in^63^ where the impulses were found using Adaptive Center-Weighted Median Filter (ACWMF)^64^ and the strong disturbances were replaced by AMF^65^. The Gaussian noise was then suppressed using BM3D and finally the previously detected weak impulses were removed using an inpainting method based on median filtering. In^66^, the AMF is first applied to find the impulsive pixels which are then replaced by the output of NLM, so that the remaining noise has approximately a Gaussian distribution. For the removal of remaining noisy pixels similar patches are grouped and then a low rank approximation with gradient regularization is applied to reconstruct the undistorted image. The ACWMF and AMF was also applied sequentially in^11^ to remove the impulses and then a method based on Laplacian scale mixture modeling and nonlocal low-rank regularization was applied.

Another approach that extends a patch-based framework was presented in Xiao et al.^67^. In this work, impulsive outliers are removed using ACWMF or AMF, depending on the assumed impulsive noise model, and the modified version of K-SVD method^68^ was applied on outlier-free pixels to finally solve an $l_1-l_0$ minimization problem. In^69^, first the pixels which are likely to be affected by the impulse noise are labeled and the image is restored considering the remaining pixels solving an optimization problem whose objective function is composed of content-dependent fidelity and a nonconvex, nonlocal low-rank regularization terms. Methods based on weighted dictionary learning, with encorporated impulsive noise detection have been also presented in^70–72^. Mixed noise removal was also considered in the problem of restoration of blurred images, in which the impulses were again detected using AMF or ACWMF and then the image was restored using variational framework based on total variation or $l_1$ norm of framelet coefficients^73–78^.

All the described above approaches remove in the first stage the impulses and then smooth out the remaining pixels. The second group of filters used for the reduction of MGIN performs the denoising in a single step due to incorporated mechanisms which detect and suppress the pixels introduced by a heavy-tailed noise. The ROAD statistic, which provides the measure of pixel corruption, was integrated within the BF in^57^. The resulting Trilateral Filter (TF) efficiently removes the impulsive noise and smooths out the Gaussian component while preserving image edges and details. Interestingly, like in^55^, a weighting function with different properties as in the standard BF was adopted, which increased the denoising capabilities of the trilateral approach. Satisfying properties of the TF caused that it was used in various denoising schemes^79^, however its efficiency decreases with the noise intensity. A fuzzy technique based on self-avoiding geodesic paths exploring the local neighborhood of pixels whose cost was defined using a fuzzy similarity measure was described in Szczepanski et al.^80^. The proposed approach is able to cope with strong MGIN, however the computational complexity of this method is very high and usually a few iterations are required to obtain satisfactory denoising results.

The method proposed in^81^ decomposes a noisy image into three key components: the ideal image, Gaussian noise, and a heavy-tailed part. To handle outliers and sparse coefficients within the ideal image, a spike and slab prior is applied. This approach is able to adaptively infer noise statistics from the training data without requiring adjustments to model hyper-parameters. Through extensive experimentation, the method has demonstrated remarkable effectiveness, particularly in the context of suppressing high-intensity mixed noise.

In the group of patch-based methods, many approaches that successfully extend the NLM algorithm^46^ were proposed. In^82^, the distances between patches are calculated considering the impulsivity of pixels expressed through the ROAD measure. This measure is also used when obtaining the weighted average of samples in the processing block which results in better efficiency when compared with the TF^57^. The authors of^83^ also used the ROAD to calculate the impulsivity of pixels and utilized the estimated pixel corruption in the construction of the fuzzy weights used in the averaging process. The methods work well for low mixed noise intensity, but fail when denoising heavily corrupted images due to the inability of ROAD to effectively detect impulses.

Another impulsiveness measure based on the median absolute deviation was utilized within the NLM framework in Xiong and Yin^84^. The NLM filter is applied twice with parameters ensuring the suppression of both impulsive and Gaussian noise. Unfortunately, this filter fails when the noise contamination is high, as the corrupted pixels tend to be preserved, otherwise the image would suffer from excessive blur. In^85^, the authors propose a NLM based procedure which first estimates the noise contamination level again using ROAD measure, then the patches from the neighborhood of the processed pixel are compared calculating a weighted sum of distances between corresponding pixels and finally the image is denoised applying a maximum likelihood estimator. Various ways of improving the efficiency of NLM were proposed in Goossens et al.^86^. Especially the influence of the choice of the weighting function on the noise smoothing efficiency was investigated. In^87^, the NLM has been combined with the region homogeneity measure, which allows to decrease the influence of impulses. Additionally, an approach exploiting the Gaussian Mixture Model whose goal was to determine the degree of similarity between image regions was used. As in the case of other approaches, this filter efficiently copes with moderate noise intensity but encounters problems when highly corrupted images need to be restored. Another restoration technique was proposed in López-Rubio^88^. In the first step the degree of pixel corruption is estimated based on Bayesian classification and then an adequate weighting of the input pixels using kernel regression framework is performed.

Recently, Convolutional Neural Networks (CNN) have been adopted to tackle the problem of image denoising. The Denoising Convolutional Neural Networks (DnCNNs), which are able to effectively exploit the global image features were used to enhance images affected by impulsive^89–91^ and Gaussian noise^92,93^. Generally, the methods based on the DnCNNs yield very good denoising efficiency^94^. However, to achieve high performance, a time-consuming training process performed on a customized dataset is needed. What is more, the information on the noise model and an estimation of the noise intensity ratio is often required, which limits their flexibility. To alleviate this problem, various blind denoising frameworks, capable of noise suppression without prior knowledge of its distribution model were proposed^95,96^. However, their effectiveness is limited in the cases of high strength of the noise contamination.

CNNs have also increasingly received attention when addressing the problem of MGIN reduction and recently, several effective methods have been introduced^97^. In^98^, the authors propose to use two CNN models divided into two steps. The first step of the blind denoising (BdCNN) is trained to remove impulsive noise and the second one to reduce the remaining Gaussian component. Another efficient algorithm of suppressing mixed noise based on CNN, which adopts computationally efficient transfer learning approach was proposed in Islam et al.^99^. In^100^, a denoising model based on Generative Adversarial Network (DeGAN), combining generator, discriminator, and feature extractor networks was designed.

A model based on variational approach and deep learning based algorithm integrated to address mixed noise removal was proposed in Wang et al.^101^. In^102^, first a deep model was trained on mixed AWGN and RVIN and then the Pixel-shuffle Down-sampling (PD) strategy was used to adapt the obtained model to real noise. The authors of^103^ proposed a convolutional blind denoising network (CBDNet) which was trained on a real-world noisy images and also on their nearly noise-free counterparts. Extensive experiments performed on various datasets demonstrated excellent performance in terms of quality metrics and subjective visual quality.

In^104^, the Multi-Stage Progressive Image Restoration Network (MPRNet) was introduced for image restoration purposes. Noteworthy performance improvements have been demonstrated by MPRNet across various datasets, with a range of image restoration challenges, including denoising, deblurring, and deraining, being effectively addressed. What sets MPRNet apart is its capacity to simultaneously handle all three types of artifacts within a unified architectural framework.

An innovative method called Noise2Void (N2V) was introduced in Krull et al.^105^. This method stands out for its unique ability to train directly on the data to be denoised, without the need for noisy image pairs or clean target images. N2V is particularly useful when dealing with images where obtaining training targets, either clean or noisy, is not feasible. While it may be intuitive to expect N2V’s performance to be limited due to the lack of training information compared to other methods, its denoising capabilities remain competitive, even without prior training. Another method based on deep neural network, capable of enhancing noise corrupted images without the need of observing the clean images was presented in Lehtinen et al.^106^.

In a recent study^107^, a novel deep learning-based method for blind image restoration was introduced. This approach formulates a comprehensive Bayesian generative model to characterize the degradation process induced by noise. Subsequently, a variational inference algorithm is employed to achieve image restoration. The method’s effectiveness is validated through experiments on blind image denoising, demonstrating its impressive performance.

In the work^108^, a framework called ResFormer, designed to enhance performance across a diverse range of testing image resolutions, including those not previously encountered, was presented. ResFormer works by processing replicated images of varying resolutions and introduces a scale consistency loss to promote interactive information exchange across different scales. Notably, it employs a global-local positional embedding strategy to smoothly adapt to input size variations, allowing it to effectively switch between different resolutions.

While deep learning algorithms bring many benefits in terms of the restoration quality, they also suffer from significant limitations. They require large datasets of diverse images that provides the same type of noise, preferably of similar intensity, that is later expected in the incoming data. Training and deploying deep learning models for real-time denoising often demands significant computational resources, which can be a drawback for resource-constrained applications. Additionally, deep learning methods may struggle with unusual or rare noise types that are not well-represented in the training data. Traditional methods with explicit noise models may perform better in these cases.

## Robust method of MGIN reduction

### Structure of the non-local means filter

The structure of the proposed filtering approach is based on the NLM framework^46,109^, which can be viewed as an extension of the BF. Let *X* be an image consisting of color pixels ${\varvec{x}}_i=\{x_i^q\}$, with $q=1,2,3$ denoting the color channel, where $i=1,\ldots , N$ is the index which describes pixels position on the image domain and *N* denotes their overall number. The BF assigns to each image pixel ${\varvec{x}}_i\in X$, the weighted average of the RGB channel values of pixels from a processing block $B_i$ of size $(2r+1)\times (2r+1)$, whose center is ${\varvec{x}}_i$. The output ${\varvec{y}}_i$ of BF depends on the spatial and radiometric similarity between ${\varvec{x}}_i$ and the neighboring pixels belonging to $B_i$

1 $$\begin{aligned} {\varvec{y}}_i= \frac{1}{Z_i} \sum \limits _{{\varvec{x}}_j \in B_{i} } w_{j,i } \cdot {\varvec{x}}_j,\qquad Z_i=\sum \limits _{{\varvec{x}}_j \in B_{i}} w_{j,i}, \quad w_{j,i}=\exp \left\{ - \rho ^2({\varvec{x}}_j,{\varvec{x}}_i) /\sigma _s^2 \right\} \cdot \exp \left\{ - {\Vert {\varvec{x}}_j-{\varvec{x}}_i\Vert }^2/\sigma _r^2 \right\} , \end{aligned}$$

with $Z_i$ being the normalizing factor and the weights $w_{j,i}$ depend on the combined spatial (topographic) and color similarity between pixels. The symbol $\Vert \cdot \Vert$ denotes the Euclidean norm in the RGB color space, $\rho$ stands for the distance between pixels on the 2D image domain, *j* and *i* are the positions of the corresponding pixels ${\varvec{x}}_j$, ${\varvec{x}}_i$ and $\sigma _s$ and $\sigma _r$ are smoothing parameters. With $\sigma _s \rightarrow \infty$, the BF approaches the Yaroslavsky Filter^110^ and for $\sigma _r \rightarrow \infty$ it boils down to the Gaussian convolutional smoothing.

As the BF output tends to exhibit some staircase effects, introduces false edges and is sensitive to noise, many improvements have been proposed^111^. Among them the NLM approach gained high popularity as it better captures the similarity (closeness) between the processed pixel and its surrounding neighbors. The NLM filter assumes the self-similarity of structures contained in a natural image and therefore dependence on the spatial distance in (1) is omitted. The radiometric (color) similarity is expressed in terms of local neighborhoods called patches or processing windows.

**Figure 1:**
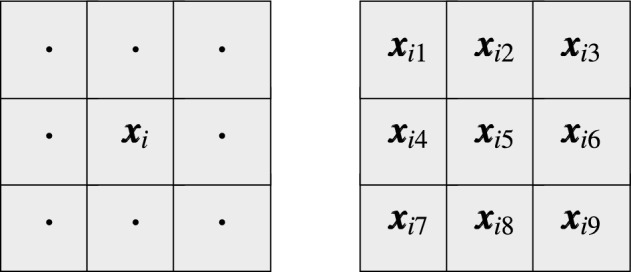
Indexing of pixels inside a window $W_i$ centered at ${\varvec{x}}_i$ with radius $s=1$. The pair of pixels which are symmetric with respect to the center pixel will be denoted as ${\varvec{x}}_{iv}$ and ${\varvec{x}}_{i\hat{v}}$. For example, if $v=4$ then $\hat{v}=6$, if $v=1$ then $\hat{v}=9$ and so forth.

**Figure 2:**
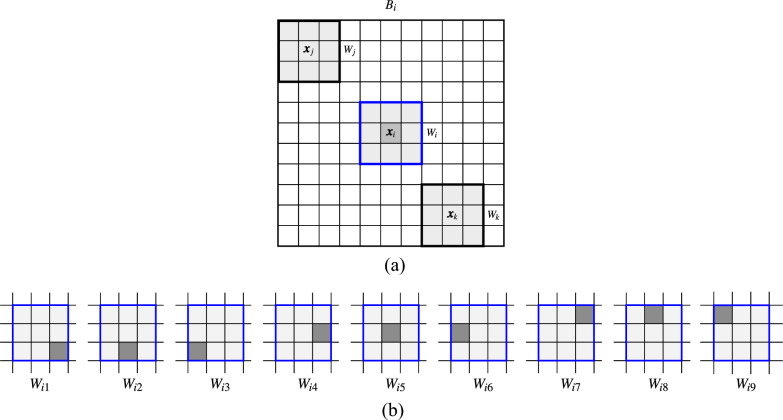
Processing block $B_i$ of radius $r=5$ centered at ${\varvec{x}}_i$ with patches $W_i$, $W_j$ and $W_k$ with size $s=1$, (**a**). The processed pixel ${\varvec{x}}_i$ (marked in dark gray) is included in the central patches $W_{iv}$ of the corresponding blocks shifted to ${\varvec{x}}_{iv}$, $(v=1,\ldots ,9)$ (**b**).

Let us define a patch of pixels denoted $W_i$ and centered at ${\varvec{x}}_i$ as a set of *n* pixels ${\varvec{x}}_{iv}$ contained in a square window of size $(2s+1)\times (2s+1)$, where $v=1,\ldots ,n$, $n=(2s+1)^2$, is an index associated with the pixels as depicted in Fig. 1. The dissimilarity measure $D(W_j,W_i)$ between two patches $W_j$ and $W_i$ centered at ${\varvec{x}}_j$ and ${\varvec{x}}_i$ is defined in the construction of NLM as the sum of squared Euclidean distances between corresponding pixels from the patches being compared, as depicted in Fig. 2a.

2 $$\begin{aligned} D(W_j,W_i)=\sum \nolimits _{v=1}^{n}\, {\Vert {\varvec{x}}_{jv} - {\varvec{x}}_{iv} \Vert }^2, \quad n=(2s+1)^2. \end{aligned}$$

**Figure 3:**
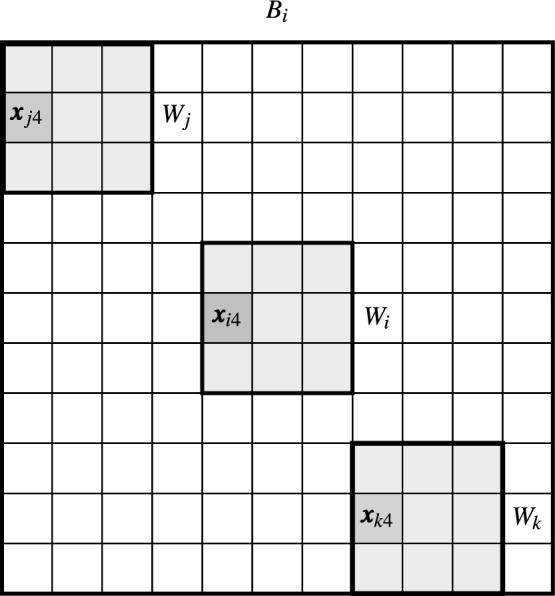
The estimate of the left pixel ${\varvec{x}}_{i4}$ from the central patch $W_i$ is computed considering the similarity to patches of the processing block.

The structure of the NLM is quite similar to that of BF formulated by (1) with the weights: $w_{j,i}=\exp \left\{ - D(W_j,W_i)/ \sigma ^2 \right\} ,$ where $\sigma$ is a smoothing parameter. The dissimilarity $D(W_i,W_j)$ can be also expressed using the Gaussian Euclidean norm, as proposed in the original definition of NLM^46^, however for small patches the Gaussian weights can be neglected. The NLM filter determines for each noisy pixel ${\varvec{x}}_i$ its estimate based on the weighted average value of all remaining pixels of the processing block $B_i$. The weights are calculated using the similarity between the patches centered at ${\varvec{x}}_i$ and pixels ${\varvec{x}}_j$ from the processing block. Such an approach is called *pixelwise*.

Another possibility is to calculate the dissimilarity measure $D(W_j,W_i)$ and assign it to each pixel in the patch $W_i$. Thus, for a pixel ${\varvec{x}}_{iv}$ belonging to $W_i$, a weighted average can be built taking the corresponding pixels ${\varvec{x}}_{jv}$ from the patches centered at pixels ${\varvec{x}}_j$ and belonging to the processing block $B_i$. Hence, we obtain estimates ${\varvec{y}}_{iv}$ of the pixels ${\varvec{x}}_{iv}$, for $v=1,\ldots ,n$, using the notation presented in Fig. 1.

3 $$\begin{aligned} {\varvec{y}}_{iv}= \frac{1}{Z_i} \sum \limits _{{\varvec{x}}_j \in B_i} w_{j,i} \cdot {\varvec{x}}_{jv}, \quad Z_i=\sum \limits _{{\varvec{x}}_j \in B_i} w_{j,i} . \end{aligned}$$

For example, the estimate ${\varvec{y}}_{i4}$ of ${\varvec{x}}_{i4}$ of patch $W_i$ will be calculated considering corresponding pixels in the patches belonging to the processing block $B_i$ as presented in Fig. 3

4 $$\begin{aligned} {\varvec{y}}_{i4}= \frac{1}{Z_i} \sum \limits _{{\varvec{x}}_j \in B_i} w_{j,i} \cdot {\varvec{x}}_{j4}\, . \end{aligned}$$

However, a given pixel ${\varvec{x}}_i$ belongs to *n* patches centered at pixels belonging to the neighborhood of the pixel ${\varvec{x}}_i$ as depicted in Fig. 2b. This offers the possibility to take the weighted average of all the estimates obtained from the collection of *n* different blocks centered at the pixels from $W_i$. This approach, called *blockwise* or *patchwise*, due to the aggregating process, proved to offer better denoising efficiency. Using this scheme, the filter output is defined as

5 $$\begin{aligned} {\varvec{y}}_i=\frac{1}{n}\sum \limits _{v=1}^{n}\frac{ \sum \limits _{{\varvec{x}}_j \in B_{iv}} w_{j,iv} \cdot {\varvec{x}}_{j\hat{v}}}{\sum \limits _{ {\varvec{x}}_j \in B_{iv} } w_{j,iv}}, \end{aligned}$$

where the block $B_{iv}$ is centered at pixel ${\varvec{x}}_{iv}$ and $\hat{v}$ denotes the index of a pixel at a position symmetric with respect to the central pixel of the patch, (see Fig. 1). The symmetric pixel is needed due to the translations of the processing block (see Fig. 2b). In this way, the filtering output is the average of *n* estimates calculated for various positions of the central pixel of the blocks $B_{iv}$ centered at ${\varvec{x}}_{iv}$ whose central patch contains the pixel ${\varvec{x}}_i$.

Yet another *global* way of aggregating the estimates, adopted in this paper, is to take the weighted average without building separate estimates, that have to be averaged and calculating the NLM output as

6 $$\begin{aligned} {\varvec{y}}_i= \frac{\sum \limits _{v=1}^{n} \sum \limits _{ {\varvec{x}}_j \in B_{iv} } w_{j,iv} \cdot {\varvec{x}}_{j\hat{v} }}{\sum \limits _{v=1}^{n} \sum \limits _{{\varvec{x}}_j \in B_{iv}} w_{j,iv} }. \end{aligned}$$

Using such an approach, the role of the small weights is diminished and only the most similar patches are being considered. Such an aggregation procedure is obviously more robust to outliers, as the corresponding weights do not influence significantly the final output. It is also possible to remove from the averaging the weights smaller than a predefined thresholding value, which can be a problem when using the approach expressed in (5), as some estimates ${\varvec{y}}_{iv}$ could not be computed in the case when the estimate is built relying on small weights, as it is the case when outlying pixels are corrupting the image. The dissimilarity measure $D(W_i,W_j)$ expressing the closeness between patches is very sensitive to outliers, which can significantly increase its value. This leads to the preservation of pixels distorted by impulsive noise, which is one of the major drawback of the NLM design.

Let us assume that the central window of the processing block *B* and some other one show high degree of similarity. If the noise distorts the central pixels of both windows and the impulses are of similar color, then the distance expressed by (2) will remain low, which will produce a large weight in the NLM averaging scheme causing that the corrupted central pixel of the block will be retained or slightly attenuated. If the impulsive noise distorts the central pixel of the block, the distances in (2) attain high values with the exception of the weight assigned to the central pixel, which is always equal to 1, as the NLM computes the similarity of the central patch to itself. This undesired effect can be alleviated omitting the central pixel when calculating the weighted average or assigning a robust estimate to the central weight, depending on the structure of the processing block and diminishing the tendency of NLM to preserve the outlying pixels. For example, the weight assigned to the central pixel of the block can be set to be equal to the highest weight calculated for all pixels in the block excluding the central one or it can be estimated using various statistical or heuristic procedures^112^, which however, can be computationally expensive.

### Robust dissimilarity measure of patches

Let $d( {\varvec{x}}_{ju}, {\varvec{x}}_{iv})={\Vert {\varvec{x}}_{ju}- {\varvec{x}}_{iv} \Vert }$ be the Euclidean distance between pixels ${\varvec{x}}_{ju} \in W_j$ and ${\varvec{x}}_{iv} \in W_i$ using the notation shown in Fig. 1, ($u, v=1,\ldots ,n$). Then the set of distances between a given pixel ${\varvec{x}}_{ju}$ and all of the pixels ${\varvec{x}}_{iv}\in W_i$ can be sorted in ascending order and we obtain a sequence of distances: $d({\varvec{x}}_{ju}, {\varvec{x}}_{i(1)} ) \le \!\dots \! \le d( {\varvec{x}}_{ju}, {\varvec{x}}_{i(k)}) \le \!\dots \! d( {\varvec{x}}_{ju}, {\varvec{x}}_{i(n)}),$ where $d( {\varvec{x}}_{ju}, {\varvec{x}}_{i(k)} )$ denotes the *k*-smallest distance between ${\varvec{x}}_{ju}$ and the pixels belonging to patch $W_i$. We propose to define the dissimilarity measure between the pixel ${\varvec{x}}_{ju}$ and the set of pixels contained in patch $W_i$ as

7 $$\begin{aligned} R({\varvec{x}}_{ju}, W_i)= \frac{1}{\alpha }\sum \nolimits _{k=1}^{\alpha } d^2\left( {\varvec{x}}_{ju}, {\varvec{x}}_{i(k)} \right) \; . \end{aligned}$$

If we take $\alpha =n$, then we calculate, the average squared distance between a pixel ${\varvec{x}}_{j,u}$ from the patch $W_j$ to all pixels of $W_i$, which is in the center of the processing block $B_i$, taking into account all pixels injected by the noise process. The outliers belonging to the patches surely influence the dissimilarity measure, so that windows from similar image regions are assigned a high value of the average squared distance between them. If $\alpha =1$, the dissimilarity measure is equal to the smallest distance between ${\varvec{x}}_{j,u}$ and pixels from $W_i$. Such a measure would be not useful, as two similar impulses injected into patches $W_i$ and $W_j$ would cause that the dissimilarity measure is low, even in the case of windows containing very differing pixel patterns.

As the window $W_i$ is assumed to be corrupted by mixed noise, we want to remove the influence of outliers and scrutinize a limited number of pixels from $W_i$, just considering only $1<\alpha <n$ pixels, which is a compromise allowing to neglect the effect of impulses, still being able to capture the similarity of the remaining, not so heavily disturbed pixels contained in the windows being compared. Calculation of the trimmed sum of distances to only $\alpha$ closest pixels has been successfully used in the construction of various filters^19^ and was also applied in the detection of outliers^113^.

The dissimilarity measures defined by (7) are calculated for all pixels belonging to $W_j$. Afterwards, their values are sorted and the color pixels from $W_j$ are correspondingly ordered: $R({\varvec{x}}_{j(1)},W_i) \le \dots \le R({\varvec{x}}_{j(k)},W_i) \le R({\varvec{x}}_{j(n)},W_i) \Rightarrow {\varvec{x}}_{j(1)} \le \dots \le {\varvec{x}}_{j(k)} \le \ldots {\varvec{x}}_{j(n)}$, where $R({\varvec{x}}_{j(k)},W_i)$ denotes the *k*-th value of the trimmed average of distances between the pixels from $W_j$ and the window $W_i$. The pixels ${\varvec{x}}_{j(k)}$, $k\in (1,\ldots , n)$, are samples generating the nondecreasing sequence of the dissimilarity measures $R({\varvec{x}}_{j(k)},W_i)$. As we are aware that the window $W_i$ may contain outliers, to make the dissimilarity robust, we consider only the set of $1 \le \beta \le n$ pixels ${\varvec{x}}_{j(1)},\dots ,{\varvec{x}}_{j(\beta )}$, which will be treated as belonging to a trimmed (pruned) window denoted as $W_{j}^{*}$. Finally, the dissimilarity measure between the windows $W_i$ and $W_j$ denoted as $\Delta (W_i,W_j)$ is defined as

8 $$\begin{aligned} \Delta (W_j,W_i) = \frac{1}{\beta } \sum \nolimits _{k=1}^{\beta } R({\varvec{x}}_{j(k)},W_i) \; . \end{aligned}$$

The distance between patches $W_i$ and $W_j$ is calculated considering only the $\beta$ pixels from $W_j$, for which the sum of squared distances to the $\alpha$ closest pixels from $W_i$ is minimized.

**Figure 4:**
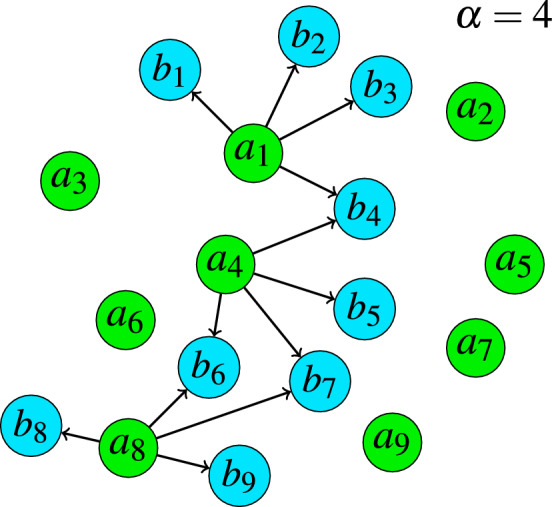
Illustration of the proposed dissimilarity measure in the 2D case. For each point $a_i \in A$, the average of $\alpha$ smallest squared distances to points $b_i\in B$, is computed. Then, the mean of the $\beta$ smallest values is determined and it serves as a dissimilarity measure between sets *A* and *B*.

**Figure 5:**
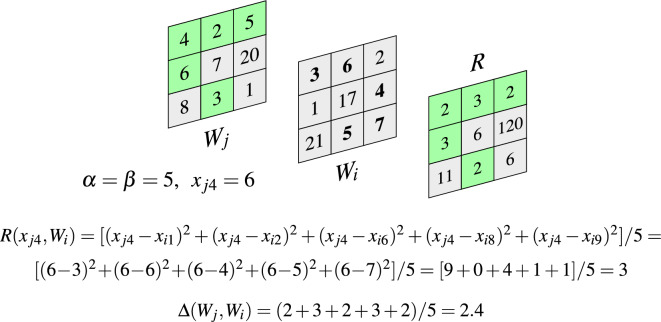
Computation of the dissimilarity measure. The pixel intensities within $W_i$ nearest to $x_{j4}$ are highlighted in bold, while pixels from $W_j$ exhibiting the highest similarity to $W_i$ are distinguished by a green color.

**Figure 6:**
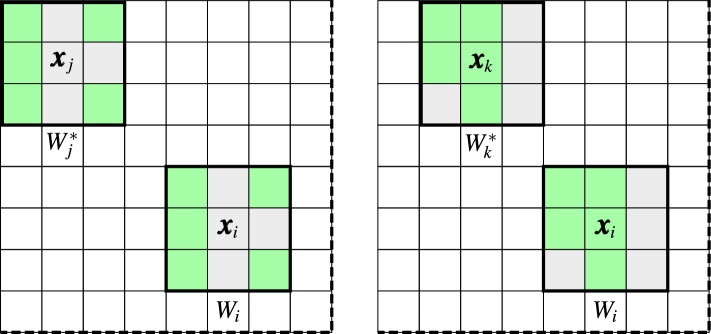
The structure of the trimmed window $W_j^*$ and $W_k^*$ centered at ${\varvec{x}}_j$, ${\varvec{x}}_k$ depends on its relation to the central window $W_i$. The green color highlights the pixels used in the averaging process, ($\beta =5$).

Figure 4 shows a simple example which explains the structure of the proposed dissimilarity measure between two sets of points *A* and *B* in 2D case. To determine the dissimilarity between *A* and *B*, first, for each point $a_i$, the distances to all points of *B* are calculated. In the example, for $a_1$, $a_4$ and $a_8$ the $\alpha =4$ closest points from set *B* are depicted. Then, for each point from set *A* the $\alpha$ smallest distances to points contained in *B* are squared and averaged. In this way, to each point $a_i$ the average of $\alpha$ smallest squared distances to points of *B* denoted as $R_i$ is assigned. These averages are sorted and the dissimilarity $\Delta (A,B)$ is the average of the $\beta$ smallest values of *R* assigned to points of set *A*.

Figure 5 presents an example which shows the way, the robust similarity measure between pixel patches is computed. First, for each pixel from $W_j$ (left) the $\alpha$ closest pixel intensities in $W_i$ (middle) is squared and averaged. The results are contained in the array *R* (right) from which $\beta$ smallest values are taken to obtain $\Delta (W_j,W_i)$. In the example the $R_4$ value assigned to the pixel $x_{j4}$ with intensity 6 in $W_j$ is shown, (the intensities of closest pixels in $W_i$ are displayed in bold). The pixels of $W_j$, which correspond to the $\beta =5$ smallest values of *R*, are marked with green color. Thus, the trimmed window $W_j^*$ is composed of the pixels marked green with intensities 4, 2, 5, 6, 3, $(x_{j1}, x_{j2}, x_{j3}, x_{j4}, x_{j8})$ and $\Delta (W_j,W_i)$ is the mean of $\beta$ smallest values of *R*: $(2+3+2+3+2)/5$.

### Structure of the proposed denoising technique

The proposed filter output of the pixels within the filtering window $W_i$ will be calculated using the patchwise global scheme defined by (6) as

9 $$\begin{aligned} {\varvec{y}}_i=\frac{1}{Z_i} \sum \limits _{v=1}^{n} \sum \limits _{ \begin{array}{c} {\varvec{x}}_j \in B_{iv} \\ {\varvec{x}}_{j\hat{v}} \in W_j^{*} \end{array} } w_{j,iv} \cdot {\varvec{x}}_{j\hat{v} } , \qquad Z_i=\sum \limits _{v=1}^{n} \sum \limits _{ \begin{array}{c} {\varvec{x}}_j \in B_{iv} \\ {\varvec{x}}_{j\hat{v}} \in W_j^{*} \end{array} } w_{j,iv}, \qquad w_{j,iv}=\exp \left( - \Delta (W_j,W_{iv})/\sigma ^2 \right) . \end{aligned}$$

This global filtering scheme will be denoted as MINOR$_{\text {G}}$. Specifically, the patchwise standard approach, as defined by Eq. (5), will be referenced as MINOR$_{\text {S}}$. It is worth noticing that the similarity of a patch to itself: $\Delta (W_i,W_i)$ is not equal to 0, which allows to use the weight assigned to the central pixel of the block in the averaging process, thus making the proposed approach insensitive to outliers, without sacrificing the ability to suppress the Gaussian noise component. Besides, special treatment of the central pixels of the processing blocks needed in the BF and NLM design aiming at removing isolated impulses can be omitted.

**Figure 7:**
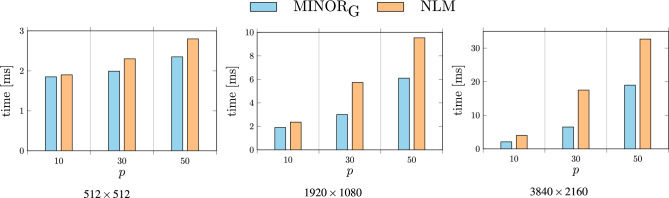
Comparison of execution times of the NLM and MINOR$_{\text {G}}$ filters with recommended parameters using images of increasing resolutions.

**Figure 8:**
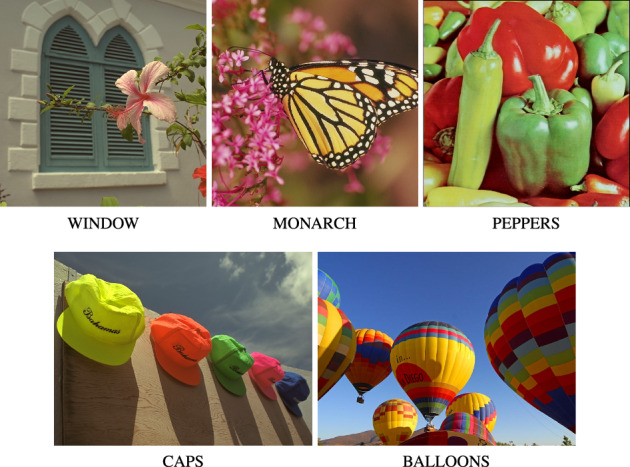
Color test images used for the experiments.

As illustrated in Fig. 6, for each patch in the block centered at pixel ${\varvec{x}}_i$ a different set of pixels is chosen as belonging to the $\beta$ most similar ones included in $W_j^*$, which generates a corresponding set of pixels from $W_i$ which are assigned a weighting value and then taken for the averaging process. It is worth noticing that also the impulses in $W_i$ will obtain their weights, as the set of pixels belonging to $W_j^*$ is not influenced directly by the outliers. Furthermore, it is advantageous that the patterns in $W_j$ and $W_i$ do not necessarily have to perfectly align, especially in the scenario of highly corrupted images where minute details become irreversibly lost.

The application of the global averaging scheme according to Eq. (6) is advantageous, as the stack of images produced in the standard NLM averaging approach usually contains corrupted pixels, whose averaging leads to the creation of noise artifacts. Using the global approach, the weights originating from noisy pixels are discarded, as they are too small to influence the final averaging result.

### Computational complexity

In the classical pixelwise NLM filter, the computation of weights assigned to each pixel within a processing block *B*, containing a total of $b=(2r+1)^2$ elements, necessitates the calculation of $n=(2s+1)^2$ squared Euclidean distances between the central patch and all other patches within the block, (see Eq. 2) . This distance computation constitutes the primary computational load of the NLM algorithm. The final output of the standard NLM filter at a given pixel involves additional steps, including the multiplication of weights with each pixel channel value, addition of the obtained values for each channel, and the calculation of the sums of weights, which are required for the division that provides the three channels of the color filter output. As a result, the computational complexity of the standard NLM filter applied to an image comprising *N* pixels is expressed as $\mathscr {O}(N\cdot n \cdot b)$^114,115^.

In the patchwise implementation, the channel values of the filter output are determined using *n* estimates, as described by Eq. (5). While the patchwise implementation is more computationally demanding, the number of required distances remains the same, and only additional processing of the weights and final averaging of the obtained *n* output images are required.

Regarding complexity, our proposed MINOR$_\text {S}$ filter, also adopting the patchwise approach, primarily differs in the calculation of weights. To determine a weight, we need to compute for every pixel in the central patch all distances to pixels in patches contained within the block, resulting in the computation of $n\cdot n$ squared Euclidean distances for each patch. Furthermore, for each pixel in the central patch, we must sort the distances to find the $\alpha$ smallest ones, followed by sorting the cumulated distances to find the $\beta$ smallest values and corresponding pixels. Excluding the sorting, the computational complexity of the proposed robust NLM is $\mathscr {O}(N\cdot n^2 \cdot b)$, which is *n* times higher than the complexity of the NLM. The MINOR$_\text {G}$ version of the new filter is faster, as all weights are multiplied with corresponding channel values and then normalized. This eliminates the need to create *n* separate estimates. Instead, the final output is computed as a sum of the weights multiplied by the channel intensities and then divided by the overall sum of weights, as shown in Eqs. (6) and (9).

It is important to note that in the proposed MINOR$_\text {G}$ approach, the required block size is significantly smaller compared to using the patchwise NLM for the same Gaussian noise intensity^109^. Consequently, even with the same level of Gaussian noise and additional impulsive noise distortion, our filter operates significantly faster and allows for real-time image processing.

Figure 7 provides a comparative analysis of the execution time for our proposed MINOR$_\text {G}$ filter, as opposed to the patchwise implementation of NLM using the parameters outlined in Table 1. The experiments were conducted using a CUDA-compatible NVIDIA RTX2080Ti graphics card, affirming the computational efficiency of our denoising approach. Notably, our proposed filter exhibits comparable or even reduced computational overhead compared to the patchwise version of NLM. This efficiency is largely attributed to the smaller processing block and patch size requirements. Processing a full HD image with a resolution of 1920$\times$1080 takes an average of just 5 milliseconds, rendering it suitable for real-time image processing applications.

**Table 1:** Recommended parameters of the patchwise NLM^109^ and the proposed MINOR$_{\text {G}}$ denoising technique.

*p*	NLM		MINOR$_{\text {G}}$			
	*s*	*r*	*s*	*r*	$\alpha$	$\beta$
10	1	10	1	1	2	5
30	2	17	1	6	4	5
50	3	17	1	12	4	5

Reducing the computational complexity, which surpasses that of the NLM filter, can be achieved by employing less computationally demanding dissimilarity measures between pixels in the patches under comparison. Moreover, integrating rapid and straightforward mechanisms to estimate the level of noise corruption within a specific patch can facilitate the exclusion of heavily corrupted patches from the averaging process, resulting in a reduction of computational workload^115^.

## Filtering results

The experiments, whose aim was to evaluate the effectiveness of the proposed filter, were performed on a database of 100 images of resolution $640\! \times 480$, which consists of color test images corrupted with various degrees of noise intensity. The dataset is available for download at http://denoising.net and also accessible as electronic supplemental material^116^. Additionally, the widely used standard color test images WINDOW, MONARCH, PEPPERS, CAPS, MOTOCROSS and BALLOONS were chosen to perform detailed examination of the proposed filter properties, (see Fig. 8).

These images were contaminated by Gaussian noise with standard deviation $\sigma _{\text {n}}\!=\!p$ and then they were corrupted by uniform impulsive noise with the percentage of corrupted pixels equal to *p*. The distortion with parameter $p=10$ means that the image was contaminated by Gaussian noise with standard deviation equal to 10 and impulsive noise with $10\%$ of corrupted pixels. Such a mixture of the impulsive and Gaussian noise was used in our previous papers and proved to simulate well the real image distortions^117,118^.

In this work, we have chosen 3 noise degradation levels $p=10$, 30 and 50. The denoising effectiveness was assessed in terms of the Peak Signal to Noise Ratio (PSNR) defined as

10 $$\begin{aligned} \text {PSNR}=10\log _{{10}}\left( {\frac{{ {255}}^{2}}{{\text {MSE}}}}\right) , \quad \text {MSE}={\frac{1}{3N}}\sum _{{i=1}}^{{N}} {\Vert {\varvec{o}}_i -{\varvec{y}}_i \Vert }^{2}, \end{aligned}$$

where ${\Vert \!\cdot \!\Vert }$ denotes the $L_2$ norm in RGB color space and ${\varvec{o}}_i$, ${\varvec{y}}_i$ stand for the original and restored image pixels. Furthermore, the Multi-Scale Similarity Measure (MSSIM) was used to better express the image restoration quality in consistency with subjective ratings^119^. In this paper this measure is presented in a logarithmic form, to allow for a more effective analysis of the results: $\text {MSSIM}_{\text {L}} =-10\log _{10}\left( 1-\text {MSSIM}\right)$.

**Figure 9:**
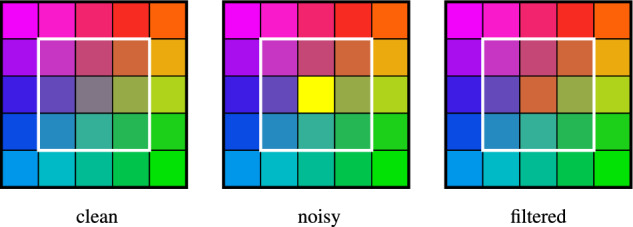
The metric IRI expresses the ability of a filter to suppress impulsive noise. If the yellow pixel in the noisy image is replaced by a pixel from the filtering window of the clean image, then the sum in Eq. (11) will be not increased. If the impulse in the filtered image is preserved, the sum will be considerably increased.

To better express the ability of removing impulses, a “relaxed” mean squared error measure is used, which leads to the Impulse Removal Index (IRI)^118,120^

11 $$\begin{aligned} \text {MSE}_{\text {R}}\!=\! \frac{1}{3N}\! \sum _{i=1}^{N}\min _{{\varvec{o}}_j\in W_i} \Vert {\varvec{y}}_i\! -{\varvec{o}}_j\Vert ^2,\quad \text {IRI}\!=\!10\log _{10}\left( \frac{255^{2}}{\text {MSE}_{\text {R}} } \right) , \end{aligned}$$

where ${\varvec{o}}_j$ are the original (clean) pixels contained in window $W_i$ and ${\varvec{y}}_i$ is the filtering output.

The IRI enables to efficiently measure the capacity of a filter to suppress the impulsive noise as shown in Fig. 9. If the impulsive pixel ${\varvec{x}}_i$ was restored, then the minimal distance between the filter output ${\varvec{y}}_i$ and the clean pixels in the window $W_i$ is determined. If the impulse is retained, no close pixel in the window of the clean image can be found, which increases the contribution to the sum in the definition of IRI. In this way, if the output of a filter is close to one of the clean pixels from the window $W_i$, small penalty is imposed on the IRI measure. In consequence, the IRI provides a reliable measure of impulsive noise suppression.

### Influence of filter parameters

In order to determine the optimal parameter values of the proposed patchwise filtering technique with global weighting as described by Eq. (9), a series of comprehensive experiments was performed. Their aim was to evaluate the influence of $\alpha$, $\beta$, $\sigma$, *r* and *s* on the efficiency of the denoising approach in terms of the commonly used PSNR quality measure.

**Figure 10:**
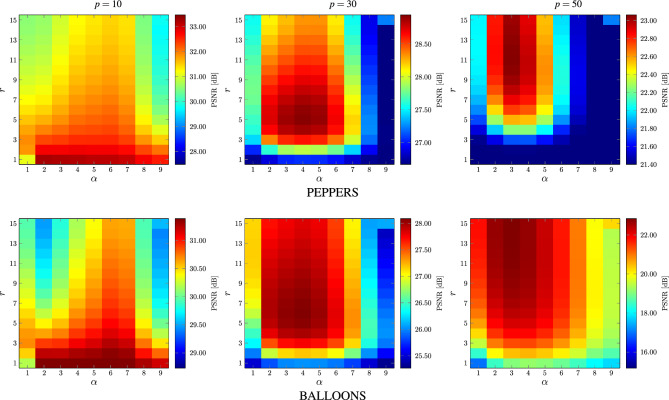
Best achievable PSNR values obtained for various *r* and $\alpha$ parameters using images depicted in Fig. 8.

**Figure 11:**
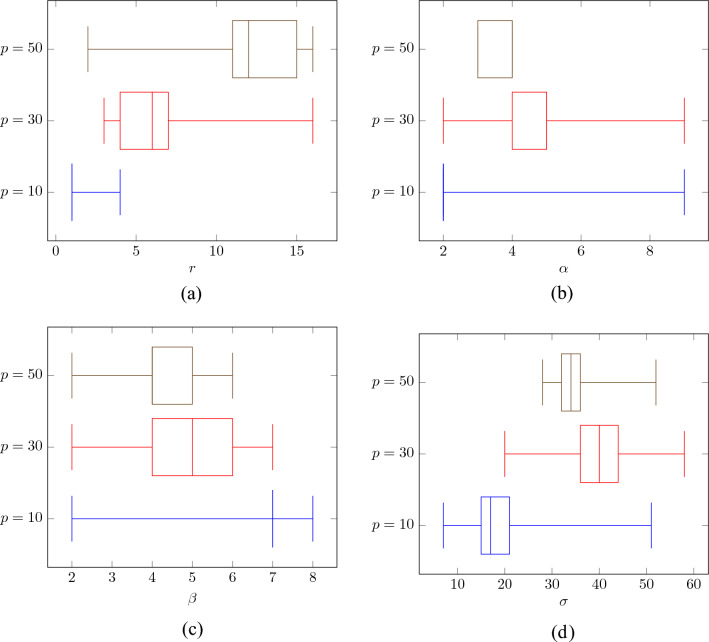
Distribution of parameters yielding the best possible PSNR values, evaluated on a set of 100 color images^116,118^, corrupted with MGIN of levels $p=10,30,50$.

**Figure 12:**
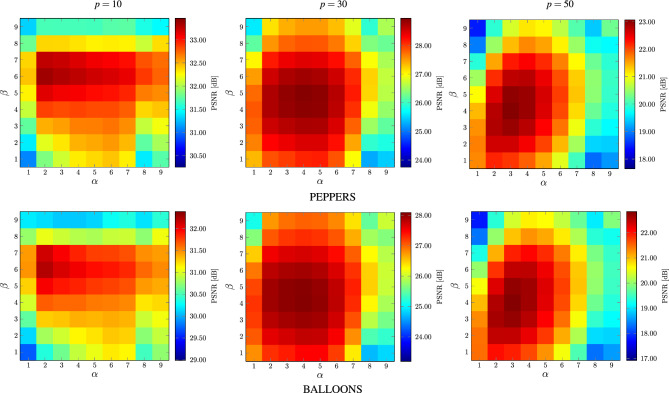
Dependence of $\alpha$ and $\beta$ parameters providing the best possible PSNR values using the images depicted in Fig. 8.

The experiments on various images contaminated with different noise intensities revealed that the patch size $3\! \times \!3$ is sufficient to obtain satisfactory denoising results for all contamination levels and the increase of *s* did not improve the quality parameters or even made it worse. Therefore, we set $s=1$ in all of the performed experiments. Then, we evaluated the impact of the radius *r* of the processing block *B* and the parameter $\alpha$ on the denoising efficiency of the proposed filter. For the analysis, exhaustive examination of all sets of parameters was performed, ($\alpha ,\beta \in [1,9]$, $r\in [1,15]$ and $\sigma \in [1,100]$ with step 1).

**Figure 13:**
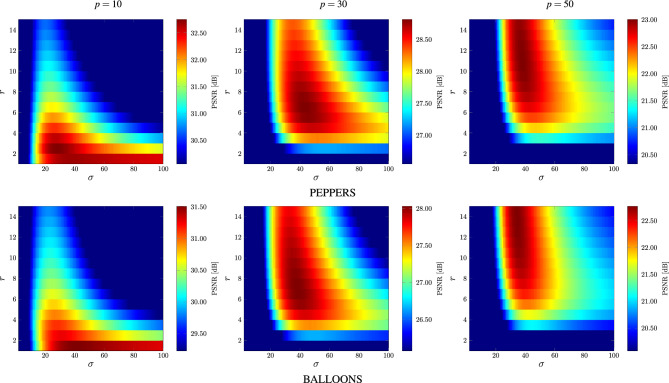
Dependence between the block size *r* and parameter $\sigma$ yielding the best achievable PSNR values.

**Figure 14:**
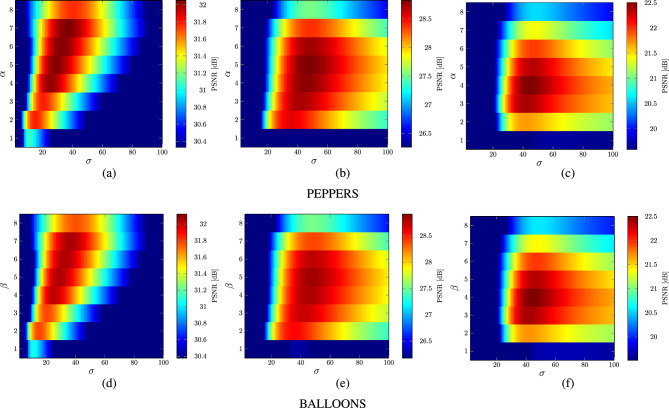
Dependence of the best possible PSNR values on the $\alpha$ and $\sigma$ when $\beta =5$ (**a**–**c**) and on $\beta$ and $\sigma$ when $\alpha =4$ (**d**–**f**) using image PEPPERS and setting block size $r=6$.

The dependence of PSNR on the radius *r* and $\alpha$ using the test image PEPPERS and BALLOONS is presented in Fig. 10. As can be observed, satisfying results were achieved for the $3\! \times \!3$ filtering block, ($r=1$) for low noise contamination ($p=10$). However, the size of the block must be increased when the noise is stronger. Good results are obtained when using $r=6$ for filtering images corrupted with noise intensity $p=30$ and $r\in [9,13]$ is needed for $p=50$. These parameter values are consistent with those obtained using a database of 100 images. The boxplot presented in Fig. 11a confirms the values of the recommended block size *r* needed to achieve best possible results in terms of PSNR.

It is worth noticing that in the classic NLM algorithm, the recommended block sizes are much larger^109^. For images distorted with Gaussian noise of standard deviation $\sigma _{\text {n}}$ less than 15, the required block size is $r=10$ and for those exceeding 30, the suggested *r* is 17. The recommended patch size is like in our algorithm $s=1$ for low contamination, however it must be increased to $s=3$ for moderate noise intensity ($30< \sigma _{\text {n}} < 45$) and even to $s=5$ for very strong pollution. It can be concluded that the required block and patch sizes needed to obtain satisfying results using our technique are significantly lower that in the case of the classic NLM, which translates directly into a significant reduction of the computational burden, which is proportional to squared *s* and *r* parameter values.

The analysis of the results depicted in Figs. 10 and 11b shows that for low contaminations the parameter $\alpha$ should be set to 2. For higher contaminations the optimal value of $\alpha$ is 4. The parameter $\beta$ is not significantly dependent on the noise intensity as depicted in Figs. 11c and 12. The setting $\beta =5$ generally yields quality measure values close to the optimal.

Finally, Figs. 11d and 13 illustrate the influence of the parameter $\sigma$ and the block size *r* on the denoising efficiency of the proposed filter for the analyzed noise contamination levels. As can be observed, the recommended value of the $\sigma$ is 20 for low intensity level and 40 for higher contaminations using the previously found optimal size *r* of the processing blocks. Figure 14 confirms that the recommended values of the filter parameters give satisfactory denoising quality evaluated through the PSNR measure. The optimal parameter values for the 3 levels of MGIN contamination are summarized in Table 1. These parameter settings were used for the comparisons of the proposed denoising approach with competitive filters.

**Table 2:** Comparison of quality measures of noisy images restored by the MINOR filter and competitive techniques.

*p*	MINOR$_\text {G}$	MINOR$_\text {S}$	FWNLM	L0TV	WESNR	NLM	RLSF	TF	PARIGI	RMMF	CROMO	ALOHA	SAW	ROMS	N2V	ResFormer	AVMR	MLSM-NLR	NLH	BdCNN	CBDNet	MPRNET	PD	WKSVD	VIRNet
PSNR																									
WINDOW																									
10	31.9	31.1	30.7	27.0	31.9	27.7	31.7	32.9	29.7	29.4	29.9	31.4	29.3	30.5	21.5	26.1	26.7	**34.4**	28.0	*33.0*	24.5	26.1	*33.5*	31.2	23.4
30	*27.3*	*27.3*	24.3	21.7	25.0	22.0	26.8	26.6	22.6	13.7	13.7	21.3	25.6	26.3	19.7	17.4	25.9	27.1	22.4	**27.5**	18.4	21.0	*27.4*	21.3	20.7
50	**22.7**	22.1	21.4	19.6	15.4	18.6	21.0	*22.2*	16.6	13.8	14.0	16.1	21.2	21.1	18.2	12.7	19.2	21.5	18.7	**22.7**	15.3	17.9	22.0	18.4	18.4
MONARCH																									
10	30.2	29.4	30.7	25.1	30.2	27.0	29.8	30.7	28.2	28.1	28.5	30.2	28.4	28.7	19.6	25.4	24.3	*31.5*	26.4	**32.7**	23.8	24.2	*31.7*	29.4	21.8
30	25.6	25.7	24.8	19.4	19.5	20.6	*25.8*	25.4	20.2	21.4	21.9	19.8	24.3	24.8	17.8	16.7	24.4	24.7	20.3	**26.3**	17.4	24.2	*25.9*	19.8	18.8
50	**21.3**	*20.7*	19.8	16.9	11.5	16.6	19.7	20.5	14.1	16.6	17.1	14.6	19.2	19.6	16.1	11.5	17.0	18.9	16.7	*20.8*	13.7	16.2	20.3	16.0	16.3
PEPPERS																									
10	33.5	33.2	32.6	27.8	31.3	27.8	33.4	*34.1*	29.3	30.7	31.1	31.5	31.9	32.7	19.8	26.5	29.2	**34.3**	27.8	29.4	23.7	26.1	*34.2*	32.3	22.1
30	*28.9*	**29.1**	26.3	22.7	24.4	20.7	28.4	28.2	23.2	23.1	23.6	19.6	25.5	27.4	17.5	16.8	27.0	26.3	20.7	28.1	17.9	20.5	*28.5*	20.1	18.9
50	**23.1**	21.8	22.2	19.5	12.5	16.5	20.6	*22.8*	16.7	16.9	17.4	14.4	20.3	20.9	15.6	12.3	16.7	19.5	16.9	*22.5*	13.8	16.8	21.2	16.2	16.0
CAPS																									
10	32.6	32.0	32.2	27.6	31.8	29.2	32.9	*34.2*	29.8	30.3	30.7	30.4	31.4	31.7	21.2	27.3	29.8	33.4	28.9	*33.5*	24.3	27.6	**34.6**	32.3	22.6
30	**29.6**	*29.5*	27.8	24.0	25.1	22.6	28.9	28.6	23.8	23.5	24.1	20.0	25.9	28.5	19.2	17.6	27.6	26.6	22.8	29.3	18.2	22.6	*29.4*	21.7	19.8
50	*24.1*	23.1	**24.7**	21.7	15.6	18.3	21.7	23.6	18.0	17.8	18.4	14.9	21.7	22.3	17.5	12.3	18.6	20.5	18.4	*24.6*	15.3	18.5	23.0	18.0	17.5
BALLOONS																									
10	32.4	31.6	31.1	27.4	29.9	27.4	*32.5*	*33.0*	28.3	30.1	30.5	31.1	26.9	31.2	18.2	25.1	27.8	**33.2**	25.4	30.2	23.5	26.1	30.4	31.4	20.9
30	**28.1**	**28.1**	25.7	22.4	21.4	19.9	*27.6*	27.3	23.1	22.8	23.4	19.6	20.0	26.7	16.5	16.2	26.2	25.1	19.2	27.2	17.2	20.2	26.7	19.1	17.5
50	**22.8**	21.3	*22.0*	19.0	11.7	15.6	20.2	*22.3*	17.3	16.8	17.3	14.2	15.7	20.5	14.7	11.6	15.7	18.8	15.4	21.8	13.9	16.0	20.5	15.3	14.9
$$\text {MSSIM}_{\text {L}}$$																									
WINDOW																									
10	*28.6*	24.1	25.7	21.2	26.9	19.2	23.6	25.7	23.6	22.1	22.9	13.1	21.8	**29.0**	11.3	18.0	22.4	*28.5*	19.4	28.2	15.7	18.9	27.7	23.9	16.1
30	**19.6**	12.1	12.1	11.3	15.6	11.1	12.9	13.5	12.5	11.7	12.1	7.4	14.8	*19.2*	8.2	7.8	16.5	17.2	11.3	*19.2*	8.4	9.4	18.5	9.7	12.6
50	**12.4**	7.1	6.3	5.3	3.5	6.6	6.4	7.2	5.3	6.2	6.4	4.5	8.4	*12.0*	5.6	3.1	7.2	8.0	5.2	10.0	4.8	4.5	*10.5*	5.4	7.5
MONARCH																									
10	25.8	24.6	26.0	19.7	26.2	21.3	23.7	25.5	23.5	22.1	22.9	24.8	25.7	26.5	13.8	18.8	23.8	*29.1*	18.2	**29.4**	14.5	20.1	*28.2*	25.9	14.1
30	*19.5*	13.1	17.7	10.7	13.8	13.3	12.7	13.5	14.1	11.6	12.0	12.2	18.6	18.6	9.9	10.1	18.6	19.3	12.5	**22.6**	8.7	*20.1*	19.3	13.1	11.3
50	*11.1*	8.7	9.3	5.9	2.3	7.7	7.2	7.8	7.9	6.5	6.8	6.8	10.5	10.3	6.8	3.7	7.6	10.4	7.3	**13.3**	5.1	6.3	*11.5*	6.9	7.9
PEPPERS																									
10	23.0	25.6	*26.3*	22.0	23.6	18.4	20.3	**27.0**	23.6	22.8	23.7	24.1	23.6	*26.1*	12.8	17.9	24.0	25.6	17.1	24.8	13.9	19.7	24.5	22.7	13.2
30	*17.7*	12.4	15.7	11.0	15.0	12.2	9.8	13.1	14.2	10.6	11.0	11.5	14.6	*18.1*	8.8	10.5	17.6	17.4	11.0	**18.2**	7.6	12.2	16.8	12.3	11.0
50	9.4	5.9	9.9	6.7	3.2	7.6	4.8	7.0	8.1	5.4	5.7	5.9	*10.5*	10.1	6.1	4.8	7.1	10.1	7.3	**11.9**	4.8	6.4	*10.8*	7.4	7.2
CAPS																									
10	**19.5**	14.3	15.7	13.2	15.2	10.6	11.2	15.8	14.8	12.9	13.4	15.3	12.9	**19.5**	7.4	8.4	13.5	17.0	8.5	*17.8*	7.5	9.8	16.6	14.2	7.0
30	**14.3**	5.7	7.9	5.1	8.0	6.5	4.9	6.5	7.2	5.4	5.7	5.8	7.0	*13.7*	4.8	4.5	8.6	8.7	5.2	*10.3*	3.9	6.2	9.6	6.1	5.0
50	**9.4**	3.7	4.8	2.7	1.7	3.8	2.2	3.1	3.2	2.6	2.8	3.0	4.5	*8.7*	3.1	2.4	3.1	4.4	3.2	*5.9*	2.3	3.8	4.9	3.7	3.8
BALLOONS																									
10	**21.9**	13.9	17.9	11.1	16.0	13.8	13.2	14.1	12.8	11.8	12.3	17.7	9.8	18.1	7.5	12.2	16.8	*19.6*	11.0	17.8	7.8	11.7	*19.7*	15.8	7.4
30	**14.8**	6.1	10.7	5.3	8.2	7.9	6.4	6.6	7.3	5.5	5.7	6.2	6.6	10.0	4.5	6.3	10.1	10.4	9.1	*14.1*	4.3	8.1	*13.2*	7.0	8.6
50	**9.0**	3.2	6.7	3.3	1.4	4.6	3.5	3.7	3.9	3.0	3.1	3.4	4.3	5.2	3.1	3.4	3.8	5.4	5.6	*7.8*	2.7	4.9	*8.0*	4.4	6.1
IRI																									
WINDOW																									
10	*30.5*	*30.5*	29.7	23.4	29.8	25.8	27.8	30.3	26.0	27.1	27.7	12.1	28.9	30.1	18.3	23.7	25.1	**32.1**	26.5	29.7	20.7	23.9	*32.0*	29.4	19.5
30	**26.2**	*25.8*	22.7	18.8	22.5	19.2	18.7	20.7	19.2	17.2	17.9	8.4	23.0	24.7	16.4	14.0	24.1	25.2	19.9	24.9	14.5	18.1	*25.7*	18.5	17.1
50	**19.9**	19.2	18.8	16.5	11.5	15.3	11.6	14.0	12.7	11.7	12.3	8.0	18.3	18.1	14.7	8.8	15.8	18.7	15.4	**19.9**	11.3	14.6	*19.3*	15.0	14.9
MONARCH																									
10	28.5	28.1	29.1	21.8	27.4	24.6	26.5	29.0	25.0	26.6	26.9	27.7	28.1	28.5	16.0	22.9	23.2	*29.4*	23.8	*29.6*	19.9	21.6	**29.8**	27.6	17.7
30	**24.8**	*24.4*	22.7	16.1	16.4	17.3	18.1	20.4	17.3	16.4	17.2	15.9	23.3	23.6	14.1	13.1	22.8	22.4	17.0	23.4	13.5	21.6	*23.7*	16.6	14.8
50	**18.8**	*17.7*	17.2	13.4	7.6	12.8	12.1	13.8	10.1	10.7	11.3	10.5	16.1	16.6	12.2	7.4	13.3	15.8	12.9	*17.7*	9.6	12.7	17.2	12.3	12.3
PEPPERS																									
10	*32.0*	*32.0*	31.5	24.1	28.0	25.2	25.6	31.3	25.6	27.9	28.6	28.9	30.9	31.5	15.8	23.4	27.1	*32.0*	25.2	26.6	19.7	23.2	**32.4**	30.1	17.8
30	**27.0**	*26.9*	24.4	19.4	20.8	16.9	15.0	21.3	19.8	16.6	17.4	15.5	22.1	25.3	13.3	12.9	24.1	23.1	16.9	25.1	13.8	16.9	*26.1*	16.3	14.7
50	**19.6**	18.1	*19.1*	15.8	8.1	12.3	8.6	14.1	12.7	10.4	11.0	10.0	16.5	17.1	11.3	8.1	12.5	15.7	12.8	*19.0*	9.5	12.9	17.6	12.0	11.8
CAPS																									
10	*32.9*	*32.8*	32.1	23.7	29.4	27.7	26.0	31.4	26.0	27.9	28.6	28.0	32.0	32.4	17.4	25.0	29.7	31.8	27.2	30.4	20.3	25.3	**34.1**	30.5	18.3
30	**28.9**	*28.5*	26.8	21.0	22.0	19.1	15.0	21.1	20.2	17.0	17.8	15.9	22.8	27.3	15.1	13.6	25.6	24.4	19.3	27.3	14.1	19.1	*28.3*	18.0	15.5
50	*20.9*	19.6	**22.2**	18.3	11.5	14.2	8.8	14.2	13.9	11.1	11.8	10.6	18.0	18.7	13.3	8.0	14.5	16.8	14.4	*21.4*	11.1	14.5	19.6	13.8	13.2
BALLOONS																									
10	*30.5*	30.0	29.7	23.6	26.0	24.2	28.0	*30.2*	24.2	27.3	27.9	28.2	23.8	29.8	14.0	21.6	25.9	**30.6**	22.0	27.4	19.3	23.1	28.4	28.6	16.4
30	**26.1**	*25.6*	23.5	18.7	17.4	15.8	19.1	20.9	19.6	16.1	16.8	15.3	16.0	*24.5*	12.2	11.9	23.1	21.7	15.1	23.6	12.9	16.4	24.0	15.0	13.1
50	**19.3**	17.4	*18.7*	15.1	7.2	11.3	11.7	14.0	13.3	9.9	10.5	9.7	11.4	16.6	10.3	7.2	11.4	14.8	11.0	*18.0*	9.4	11.8	16.6	10.9	10.5

The top three results are distinguished in bold and italics, with the best result presented in bold.

### Comparison with competitive denoising techniques

The evaluation of the new filter’s performance was performed using a set of the well known filtering methods with parameter settings suggested in the respective papers describing them. These filters are listed below:

- Fuzzy Weighted Non-Local Means method, (FWNLM)^83^,
- TV-based restoration with $\ell _0$TV-norm data fidelity, ($\ell _0$TV)^121^,
- Weighted Encoding with Sparse Nonlocal Regularization, (WESNR)^71^,
- Non-Local Means, (NLM)^109^,
- Robust Local Similarity Filter, (RLSF)^117^,
- Trilateral Filter, (TF)^57^,
- Patch-based Approach to Remove Impulse-Gaussian NoIse, (PARIGI)^85^,
- Restricted Marginal Median Filter, (RMMF)^122^,
- Combined Reduced Marginal Ordering, (CROMO)^123^,
- Annihilating LOw-rank HAnkel matrix filter, (ALOHA)^124^,
- Self-Avoiding Walks based filter, (SAW)^80^,
- RObust Mean Shift, (ROMS)^118^.
- Noise2Void-learning denoising from single noisy images, (N2V)^105^
- ResFormer:Scaling ViTs with Multi-Resolution Training, (ResFormer)^108^
- Adaptive Variational Method for Restoring color images, (AVMR)^37^
- Modified Laplacian Scale Mixture Modeling and Nonlocal Low Rank approximation, (MLSM-NLR)^11,59^
- NLH: A blind pixel-Level non-local method for real-world image denoising, (NLH)^51^
- Blind Denoising of Mixed Gaussian-impulse Noise by Single CNN, (BdCNN)^98^
- Convolutional Blind Denoising Network, (CBDNet)^103^
- Multi-stage Progressive image Restoration NeTwork, (MPRNeT)^104,125^
- Pixel-shuffle Down-sampling Denoising, (PD)^126^
- Weighted KSVD, (WKSVD)^127^
- Variational Image Restoration Network, (VIRNet)^107^

**Figure 15:**
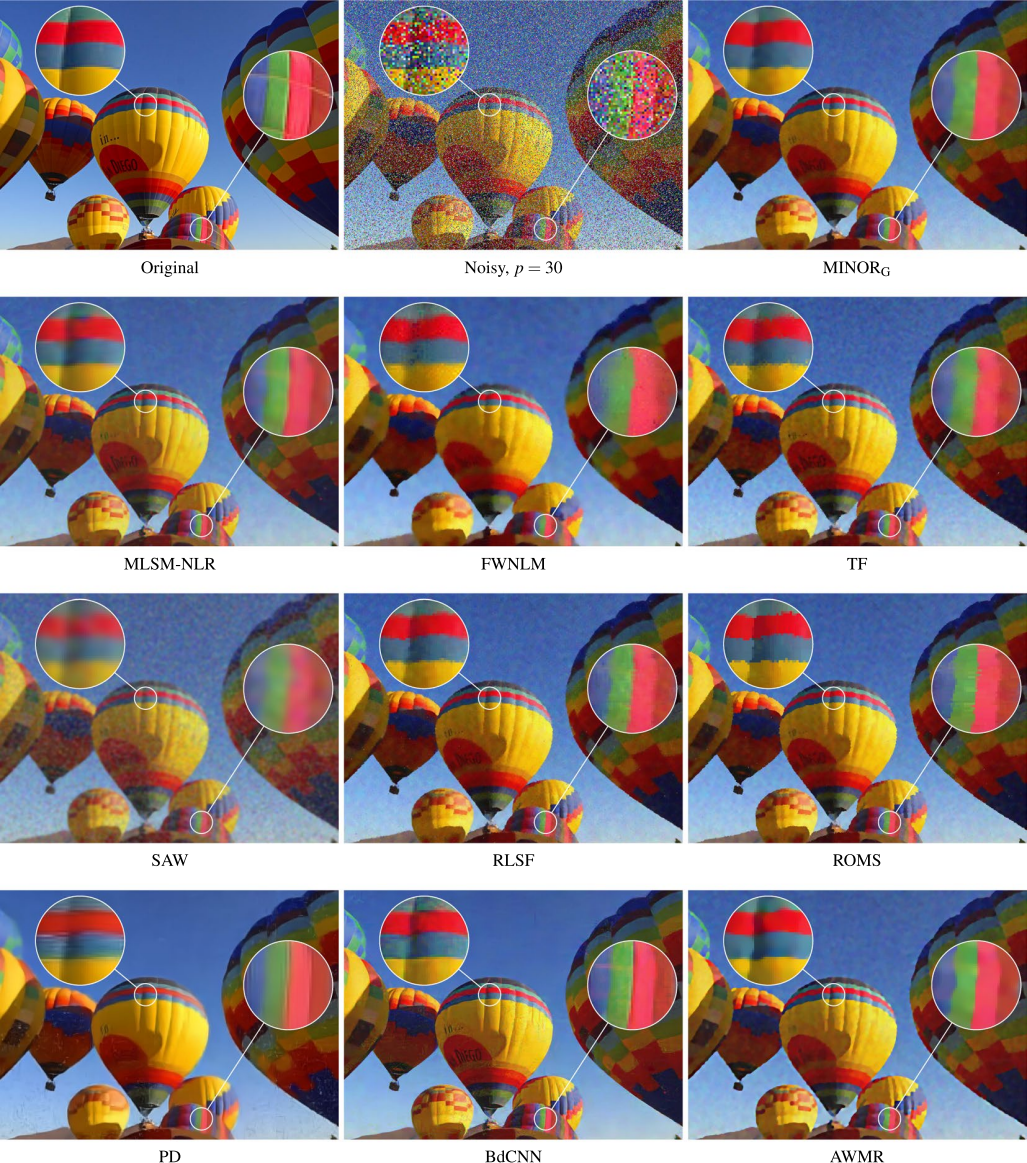
Efficiency of the evaluated filters when using the BALLOONS test image contaminated with MGIN of $p=30$.

**Figure 16:**
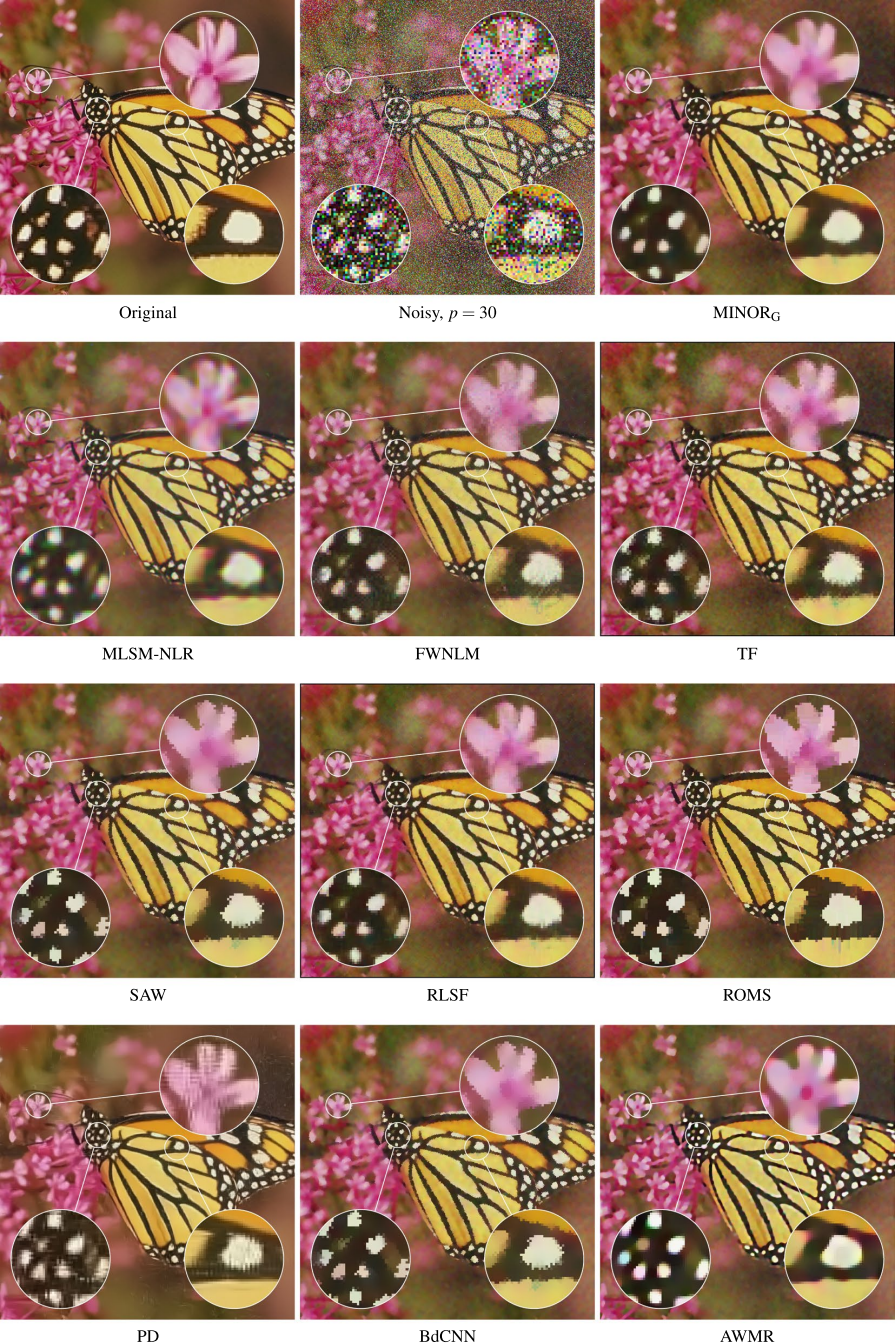
Efficiency of the evaluated filters when using the MONARCH test image contaminated with MGIN of $p=30$.

**Figure 17:**
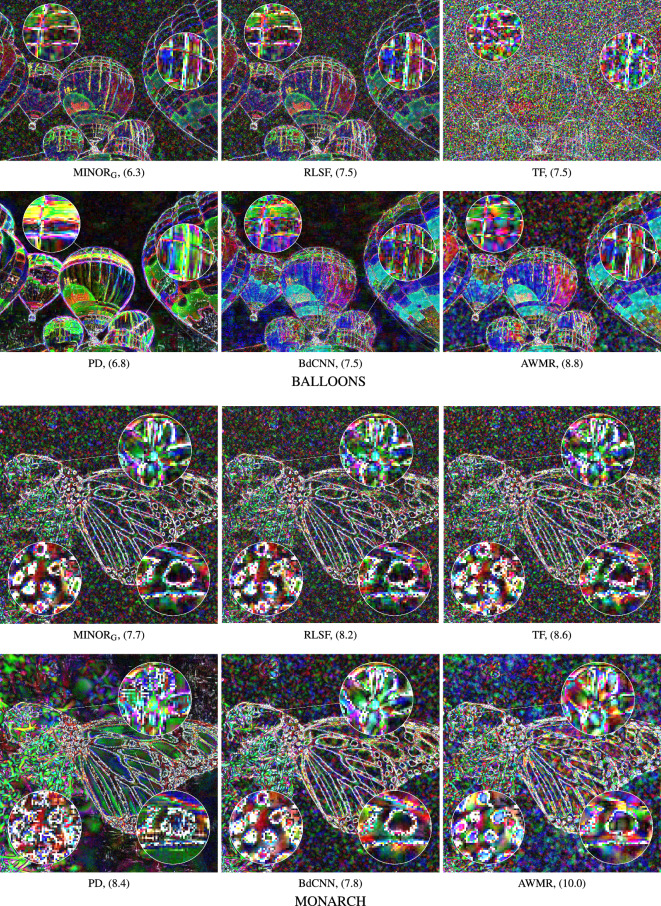
Error maps of the most efficient filters when using the BALLOONS and MONARCH test images contaminated with MGIN of $p=30$. The MAE values are enclosed in brackets.

We compared the proposed MINOR filter using the *global* averaging, (indicated by the subscript G - MINOR$_{\text {G}}$), according to Eq. (6), and using the standard *blockwise* implementation, (denoted as MINOR$_{\text {S}}$), which performs the averaging of a stack of $n=(2s+1)^2$ images. As can be seen, the global averaging provides slightly better results in terms of PSNR for higher contaminations. However, when scrutinizing the MSSIM$_{\text {L}}$ and IRI measures, MINOR$_{\text {G}}$ performs better also for images contaminated with low intensity noise.

Upon scrutinizing the Table 2, which encapsulates the PSNR, MSSIM$_{\text {L}}$, and IRI measures across a range of 23 filters, noteworthy observations emerge. Specifically, the MLSM-NLR, TF, PD, WKSVD, and BdCNNN filters demonstrate high efficiency, particularly evident in their performance with low-contamination images. However, it is noteworthy that the proposed MINOR filter consistently ranks among the top 5 methods, irrespective of the test image or the quality metric applied. Notably, when confronted with higher contamination levels, our proposed denoising approach consistently yields the most favorable objective quality measures. It notably outperforms competitive filters, particularly excelling in terms of the IRI index.

Figures 15 and 16 present a comparative analysis of filtering outcomes achieved with the BALLOONS and MONARCH test images, both contaminated by noise at an intensity level of $p=30$. The MINOR$_{\text {G}}$ filter, utilizing the recommended parameters, demonstrates favorable results in preserving edges and suppressing noise within homogeneous image regions. However, its limitation lies in the challenge of preserving intricate details in slightly distorted images, attributed to the design of the dissimilarity measure applied to patches, which does not account for the spatial pixel arrangement. Nevertheless, this specific design is advantageous for the restoration of heavily degraded images where fine details are typically lost.

The conducted experiments demonstrate that the suggested algorithm efficiently accomplishes denoising in a single step when using the recommended parameter values. Additional denoising attempts, even with adjusted parameters $\alpha$ and $\sigma$, result in excessive image smoothing. However, for the highly distorted image at $p=50$, as anticipated, a second iteration effectively eliminates residual noise and enhances denoising performance. Given that the filter executes denoising in a single pass, its computational complexity is akin to methods employing local averaging, such as NLM, TF, FWNLM, and RLSF.

Figure 17 displays color error maps, showcasing the discrepancies between the restored and clean images. These error maps represent the absolute differences between corresponding color components of the compared images, each value multiplied by a factor of 10 to enhance visibility. The pixel intensity within the error map corresponds to the accuracy of color restoration, where darker regions indicate accurately recovered pixels, while brighter regions signify poorly restored pixels. The hue in these regions is indicative of the channel that was most poorly reconstructed. Furthermore, the values of the Mean Absolute Error (MAE)

12 $$\begin{aligned} \text {MAE}={\frac{1}{3N}}\sum _{{i=1}}^{{N}} \sum _{q=1}^{3} \left| {\varvec{o}}_i^q -{\varvec{y}}_i^q \right| , \end{aligned}$$

widely used for assessing image detail preservation, are also provided, (${\varvec{o}}$ and ${\varvec{y}}$ denote the original and denoised images).

The competitive filters often exhibit tendencies to either overly smooth noisy images or leave residual blotches and incorrectly suppressed impulses. When addressing heavily corrupted images, notably successful denoising outcomes were achieved using deep learning-based approaches, such as PD and BdCNN. Figure 17 illustrates the discernible preservation of image details and effective noise suppression in smooth image regions, highlighted by the darker areas in the error map of the BALLOONS test image. However, the performance of the deep learning based filters is contingent upon the structure of the images, occasionally resulting in visible artifacts, especially at edges.

The error maps, alongside the MAE values, affirm the strong denoising capability of the proposed filtering framework. It is important to highlight that the MINOR$_\text {G}$ method effectively filters the test images with a contamination level of $p=30$, showcasing the lowest MAE values and signifying its high capacity to preserve image details.

**Figure 18:**
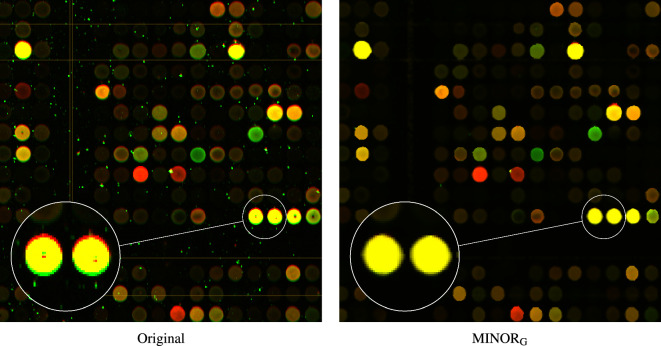
Filtering efficiency of the proposed MINOR$_{\text {G}}$ filter when denoising a noisy cDNA image. The artifacts caused by misalignment of the spots are successfully removed.

**Figure 19:**
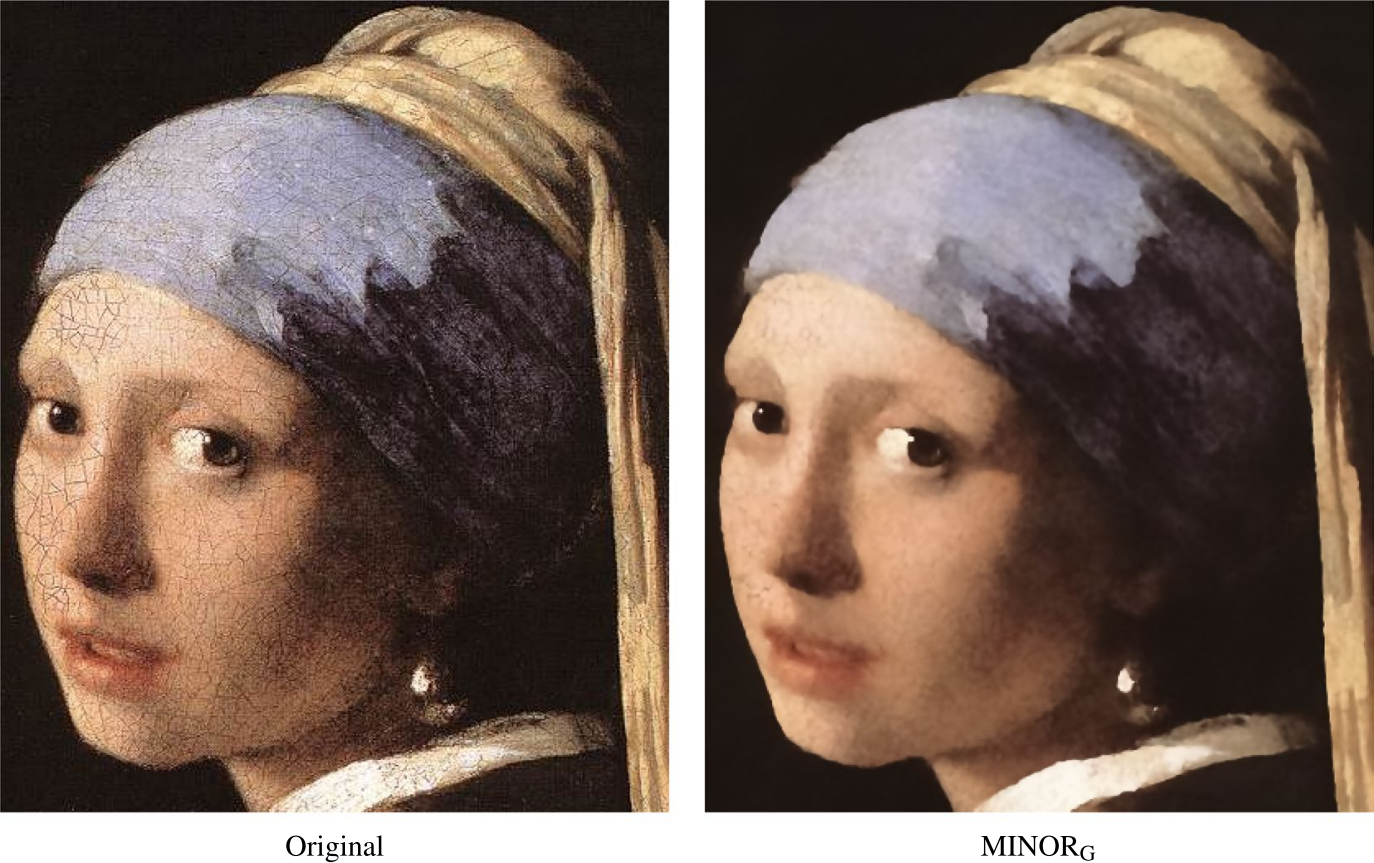
Filtering efficiency of the proposed MINOR$_{\text {G}}$ filter when performing a virtual restoration of the painting “Girl with a Pearl Earring” by J. Vermeer.

The performance of the MINOR method can be also assessed when filtering real noise contamination in cDNA image which determines gene expression levels^128^. As can be observed in Fig. 18, impulsive noise is removed, edges are well restored and the visible color artifacts, resulting from incorrect alignment, are eliminated. The beneficial properties of the new filter are also illustrated when denoising a part of the painting “Girl with a Pearl Earring” by J. Vermeer, (Fig. 19). The painting cracks are removed, no color speckles are created in the painting’s homogeneous areas and the details are well preserved.

## Conclusion

In this paper, a novel technique intended for the reduction of high intensity mixed Gaussian and impulsive noise in color images has been proposed. The elaborated approach is based on the novel dissimilarity measure between image patches, incorporated into the design of the Non-Local Means technique. The new filter discards from each patch the pixels with the highest dissimilarity to the central window of the processing block, which ensures the suppression of impulsive noise and enables to smooth out the Gaussian noise component. Thus, for each patch in the block, the most similar pixels to the central patch are determined and used for blockwise NLM smoothing. The proposed approach is well suited for highly contaminated images, whose corrupted pixels should be excluded from the averaging process. Additionally, we observed that for high noise contamination level, the classic approach used in NLM, which is based on averaging of a stack of images created from corresponding pixels from the local patches, does not fully exploit the potential of the proposed framework, especially in terms of visual quality and the ability to remove the impulsive noise. Better denoising efficiency is achieved when averaging globally all pixels in the block. In this way, the role of the low weights assigned to corrupted pixels is diminished and the final restoration result is composed of pixels which were not significantly affected by the mixed noise.

The comparison with a variety of filters intended for the suppression of mixed noise in color images revealed the satisfactory properties of the proposed denoising framework, especially at high contamination levels. The efficiency of the new filter was confirmed on a large set of test color images and was also verified when denoising images corrupted by noise of unknown structure. The computational efficiency of the proposed filter is moderate, comparable to NLM, allowing for its implementation in real-time scenarios.

Future work will be focused on the refinement of the proposed dissimilarity between image patches by taking into account the measure of pixel corruption evaluated using digital paths exploring the local neighbourhood of pixels. Additional effort will be devoted to the elaboration of an adaptive technique, which will automatically adjust the two parameters of the novel dissimilarity index, so that the filter will be able to tune its parameters to local image and noise structures.

The source code is available for download at https://github.com/dkusnik/MINOR.
